# Extracellular Matrix Ligand and Stiffness Modulate Immature Nucleus Pulposus Cell-Cell Interactions

**DOI:** 10.1371/journal.pone.0027170

**Published:** 2011-11-07

**Authors:** Christopher L. Gilchrist, Eric M. Darling, Jun Chen, Lori A. Setton

**Affiliations:** 1 Department of Biomedical Engineering, Duke University, Durham, North Carolina, United States of America; 2 Department of Molecular Pharmacology, Physiology, and Biotechnology, Center for Biomedical Engineering, Brown University, Providence, Rhode Island, United States of America; 3 Department of Orthopaedics, Brown University, Providence, Rhode Island, United States of America; 4 School of Engineering, Brown University, Providence, Rhode Island, United States of America; 5 Department of Orthopaedic Surgery, Duke University Medical Center, Durham, North Carolina, United States of America; Ohio State University, United States of America

## Abstract

The nucleus pulposus (NP) of the intervertebral disc functions to provide compressive load support in the spine, and contains cells that play a critical role in the generation and maintenance of this tissue. The NP cell population undergoes significant morphological and phenotypic changes during maturation and aging, transitioning from large, vacuolated immature cells arranged in cell clusters to a sparse population of smaller, isolated chondrocyte-like cells. These morphological and organizational changes appear to correlate with the first signs of degenerative changes within the intervertebral disc. The extracellular matrix of the immature NP is a soft, gelatinous material containing multiple laminin isoforms, features that are unique to the NP relative to other regions of the disc and that change with aging and degeneration. Based on this knowledge, we hypothesized that a soft, laminin-rich extracellular matrix environment would promote NP cell-cell interactions and phenotypes similar to those found in immature NP tissues. NP cells were isolated from porcine intervertebral discs and cultured in matrix environments of varying mechanical stiffness that were functionalized with various matrix ligands; cellular responses to periods of culture were assessed using quantitative measures of cell organization and phenotype. Results show that soft (<720 Pa), laminin-containing extracellular matrix substrates promote NP cell morphologies, cell-cell interactions, and proteoglycan production *in vitro*, and that this behavior is dependent upon both extracellular matrix ligand and substrate mechanical properties. These findings indicate that NP cell organization and phenotype may be highly sensitive to their surrounding extracellular matrix environment.

## Introduction

The nucleus pulposus (NP) is the central, gelatinous region of the intervertebral disc (IVD) which contributes to the tissue's mechanical function by resisting and redistributing spinal compressive loads. In the developing and immature human IVD, the NP is populated by cells derived from the embryonic notochord [1], [2], [3], [4], which are thought to play both biosynthetic [5], [6], [7] and stimulatory [6], [8], [9] roles in the production and maintenance of a hydrated (i.e. proteoglycan-rich), mechanically-functional NP tissue. These notochordally-derived immature NP cells exhibit several morphologic features which reflect their unique embryologic origin and distinguish them from other cells in the disc: they are large, highly vacuolated cells [10], [11], [12], [13] and are arranged in cell clusters with strong cell-cell interactions [10], [11], [14],[15],[16]. With age, however, this cell population is altered or disappears (as identified morphologically) in humans, with only a sparse population of chondrocyte-like cells remaining in the adult NP [10], [17]. This change appears to precede or coincide with structural changes in the disc, including loss of disc height and decreased proteoglycan and water content [17], [18], [19], [20], and thus alterations to this cell population may be an initial event contributing to eventual disc degenerative changes [21]. An understanding of the biology of these cells and their changes with aging may yield insight into disc degeneration mechanisms and facilitate the development of NP tissue regeneration strategies [8], [22], .

Cell-cell interactions and cell morphology are known to regulate a number of cell processes, including cell survival, metabolic activity, and the control of cell phenotype [26], [27], [28]. Environmental cues provided by the extracellular matrix (ECM) are critical regulators of cell-cell interactions, with both ECM ligand and mechanical stiffness shown to be important variables affecting cell organization and function [29], [30], [31], [32], [33]. Immature NP cells reside within a soft, gelatinous extracellular matrix environment [34], [35], and our previous studies have identified that this environment is rich in several isoforms of the ECM ligand laminin, which NP cells interact with via integrin and non-integrin laminin receptors [13], [36], [37]. The presence of these laminin ligands and receptors appears to be unique to the NP region of the disc, and their pattern of expression may be altered with aging and degeneration [36]. How these specific ECM environmental cues (mechanical stiffness, cell-ligand interactions) modulate immature NP cell organization or phenotype is not well understood.

The objective of this study was to investigate the role of the ECM environment on immature NP cell organization and phenotype. This study's primary hypothesis was that culturing immature NP cells on soft, laminin-rich basement membrane gel substrates would promote NP cell-cell interactions, morphologies, and phenotypes similar to those observed in immature NP tissues. A secondary hypothesis was that NP cell responses observed on these gel surfaces are modulated by ECM ligand, substrate stiffness, or both. These hypotheses were investigated by culturing immature NP cells on polyacrylamide gel substrates with tunable mechanical properties and functionalized with specific ECM ligands, including laminin-rich basement membrane extract, and type II collagen, another ECM ligand present in the NP. Our results indicate that soft, laminin-rich substrates (but not type II collagen substrates) promote NP cell-cell interactions and 3D morphologies similar to those observed *in situ* and that these behaviors are associated with increased levels of proteoglycan production, a key measure of immature NP cell phenotype.

## Materials and Methods

### Cell isolation and culture

Lumbar spines from skeletally immature pigs (3–6 months old, from local abattoir (Nahunta Pork Center, Raleigh, NC)) were obtained within 8 hours post-sacrifice. Cells were isolated from the NP tissues via pronase-collagenase enzymatic digestion [38], then resuspended in culture media (Ham's F-12 media (Gibco, Invitrogen, Carlsbad, CA, USA) supplemented with 5% FBS (Hyclone, Thermo Scientific, Rockford, IL, USA), 10 mM HEPES (Gibco), 100 U/mL penicillin (Gibco) and 100 mg/mL streptomycin (Gibco)). NP tissues from this source have previously been shown to be rich in cells with a characteristically notochordal cell morphology (80–90%) [13], [22]. Resuspended cells were seeded immediately onto culture substrates and cultured for up to 12 days under hypoxic conditions (5% O_2_, 5% CO_2_, 37°C), with culture media exchanged every 3 days. For cell mechanical characterization (via atomic force microscopy (AFM), as described below), freshly isolated NP cells were seeded onto tissue culture plastic surfaces for immediate (within 2 hours) testing.

### Gel culture substrates

Thin layer gel substrates of basement membrane extract (BME) were created by dispensing 90 µL of ice-cold, unpolymerized BME solution (Trevigen, Inc; growth factor-reduced, 13.8 mg/mL) into 12 mm diameter wells (custom polydimethylsiloxane molds on glass coverslips) and allowed to gel for 30 minutes at 37°C in a humidified incubator. Resulting gels were approximately 500 µm in thickness. BME is a solubilized basement membrane preparation extracted from the Engelbreth-Holm-Swarm (EHS) mouse sarcoma tumor, which contains high concentrations of several ECM proteins: laminin-111 (∼60%), type IV collagen (∼30%), entactin (∼8%), and heparin sulfate [39].

Polyacrylamide gel substrates with defined mechanical properties were created by polymerizing acrylamide with varying amounts of bis-acrylamide crosslinker [40]. An acrylamide solution (5 or 8% final concentration, mixed from a 40% stock solution; Bio-Rad, Hercules, CA) was mixed with bis-acrylamide (0.02–0.15%, from 2% stock solution, Bio-Rad) to create substrates of different stiffnesses. Solutions were degassed (20 min under vacuum in degassing sonicator bath (Branson B1510, Danbury, CT)), and polymerization initiated by adding 10% ammonium persulfate (1∶200, Bio-Rad) and n,n,n′n′-(tetra)ethylenediamine (TEMED, 1∶2000, Bio-Rad). Thin gels were formed by pipetting 10 µL of polymerizing acrylamide solution onto aminosilanated glass coverslips (22 mm square coverslip treated with 3-aminopropyltrimethoxysilane; Sigma, St. Louis, MO), and immediately covering the drop with a hydrophobic coverslip (12 mm-diameter glass treated with Rain-X, SOPUS Products, Houston, TX). Gels were allowed to polymerize for 30 minutes at room temperature, then submersed in buffer (50 mM HEPES, pH 8.0, Gibco) for 3 minutes, and top coverslips were removed with a fine forceps. Polyacrylamide gels were stored (up to 1 week) in HEPES buffer at 4°C until use. Gel thickness was determined by mixing fluorescent microspheres (2 µm-diameter Fluospheres, Molecular Probes; nile red fluorophore, Ex/Em: 535/575 nm) in gel solution prior to polymerization, with thickness measured via confocal microscopy (Zeiss LSM 510, 63× water immersion objective, NA = 1.2, Carl Zeiss USA, Thornwood, NY).

Polyacrylamide gels were functionalized to permit NP cell adhesion by covalently linking specific ECM ligands to gel surfaces (non-functionalized gels did not support any cell adhesion). Ligands were coupled by first reacting gels with a UV-activated heterobifunctional crosslinker (Sulfo-SANPAH, Pierce, Rockford, IL; 0.5 mg/mL in 50 mM HEPES, pH 8.5) under UV light (365 nm, 8 min exposure). The crosslinker solution was removed and the procedure was repeated a second time. Gels were washed twice with cold HEPES buffer (pH 8.5) to remove unreacted crosslinker and then incubated with ECM ligand (overnight at 4°C on shaker plate). For this experiment, two ligands were evaluated: (1) unpolymerized BME (diluted to 200 µg/mL in cold 50 mM HEPES w/5 mM EDTA, pH 8.5 to prevent BME self-polymerization), or (2) collagen type II (Sigma; 200 µg/mL in 50 mM HEPES, pH 8.5), an abundant ECM ligand in the NP [41], and to which NP cells are known to adhere [13], [42]. Polyacrylamide gels were washed twice with sterile PBS (pH 7.4) prior to cell seeding.

### Mechanical characterization of gel substrates and cells

Micro-scale mechanical properties of gel substrates (polyacrylamide and BME gels) were measured via atomic force microscopy (AFM) indentation (MFP-3D; Asylum Research, Santa Barbara, CA). Cantilevers (nominal stiffness = 60 pN/nm, actual stiffness determined via thermal calibration; Novascan Technologies Inc.) with 5 µm-diameter borosilicate spherical-tipped probes were used to indent gel substrates with a constant indentation rate of 15 µm/sec. The resulting force-indentation curves were fit to the Hertz contact model for spherical indentation of a flat surface [43]. A Poisson's ratio of 0.45 was assumed based on macroscopic tension tests performed previously [44]. Probe-surface contact was identified using the Contact Point Extrapolation (CPE) method [45], with the Young's modulus, E, determined from the slope of *F^2/3^* versus δ (where *F* = normal indentation force, δ = indentation). For each gel formulation, a total of 75 indentation tests (5×5 grid of points spaced 10 µm apart at 3 separate locations) were performed. Elastic moduli were compared among substrates using a one-factor ANOVA (substrate) and Tukey's HSD. Gel elastic moduli were also measured before and after functionalization with BME ligand for one gel formulation (5% acrylamide, 0.1% bis-acrylamide) to test whether gel stiffness was altered by ECM functionalization (differences assessed via Student's t-test).

The micro-scale mechanical properties of NP cells were also measured via AFM indentation to compare cell mechanical properties with those of gel substrates. Porcine NP cells were seeded (10,000 cells/cm^2^, 5% FBS culture media) on plastic petri dishes (0.1% gelatin-coated, 35 mm diameter, Becton Dickinson) and allowed to attach for 2 hr (37°C, 5% CO_2_) prior to testing, which was the minimum time necessary to permit attachment of freshly isolated cells. Attached cells remained rounded and had not begun to spread, approximating the rounded morphology of NP cells observed *in situ* [46]. Cells were indented with a 5 µm spherical-tipped AFM cantilever using the protocol and data analysis methods described above, except that a Poisson's ratio of 0.49999 was assumed for cells [47]. NP cells were large enough in size to be indented at 3 different locations per cell. Elastic modulus and cell height (measured using the difference between indentation contact point on the cell and that of the adjacent substrate) were measured for n = 30 NP cells. The correlation between cell height and elastic modulus was examined using a linear least-squares regression analysis (p<0.05 considered significant for all tests).

### Analysis of cell organization

NP cells were seeded onto gel substrates (18,000 cells/cm^2^) and cultured for up to 7 days. Cells on gel substrates were washed once with DPBS (Dulbecco's PBS with Ca^2+^ and Mg^2+^, Gibco) and fixed with 4% formaldehyde (Electron Microscopy Sciences, Hatfield, PA; diluted in DPBS) for 10 minutes at room temperature. Cells were then permeabilized (0.1% Triton X-100, Sigma), washed with DPBS, and labeled for actin (Alexa 488 phalloidin, Invitrogen) and cell nuclei (propidium iodide, Sigma).

Cells were imaged via confocal microscopy to evaluate cell organization (numbers of cells arranged in multi-cell clusters) and dimensions of cell clusters. The percentage of cells present in discrete clusters (defined as groups of >4 cells in contact with each other, separate from other cells) was quantified by counting cell nuclei using image analysis software (NIS-Elements BR, Nikon, Melville, NY). Since clusters were found to be 3-dimensional (often >5 cell layers high) and individual cell nuclei within clusters could not always be distinguished, an image analysis method was developed to estimate cell numbers within clusters. Confocal image stacks of clusters and individual cells were acquired (20×, 0.5NA objective; each confocal slice = 460 µm×460 µm×6.7 µm), with the slice thickness (6.7 µm) chosen to approximate the diameter of a cell nucleus. Single cell nuclear signal was determined by selecting individual (non-clustered) cell nuclei within an image and summing the fluorescent signal from all image slices, yielding an average fluorescent signal per individual cell. For each cell cluster, a volume containing the cluster was outlined and fluorescent signal intensity within the volume summed to give fluorescent signal per cluster. Following background intensity corrections, cluster signal was divided by single cell signal to yield an estimate of cell number per cluster. The method does not account for possible nuclear intensity differences between single cells and those in cell clusters; however, the technique was verified for several cell clusters via manual counting, with differences always less than 15%. For analysis of cell clustering, the percentage of cells present in clusters was calculated for each image field of a given ligand-stiffness substrate condition (n = 4–7 image fields per condition from 2–3 separate cell isolations, 20–70 clusters per substrate condition, >3000 cells per condition counted), with differences in clustering percentage detected using two-factor ANOVA (ECM ligand, substrate stiffness) and Tukey's HSD.

Cell cluster dimensions including height (perpendicular to gel surface), area (maximum area projected onto gel surface), and maximum dimension (in-plane with gel surface) were measured from confocal image stacks of fluorescent actin label (20×0.5NA objective, 6.7 µm confocal slice thickness) using image analysis software (Zeiss LSM). Cluster dimensions were analyzed for each substrate-ligand condition (n = 25 clusters per condition, from 2–3 separate cell isolations), with differences detected amongst conditions via ANOVA and Tukey's HSD.

### sGAG production

NP cell production of sulfated glycosaminoglycans (sGAGs) was analyzed using the dimethylmethylene blue (DMMB) spectrophotometric method [48]. NP cells were seeded onto culture substrates (88,000 cells/cm^2^) and cultured for up to 12 days, with total sGAG assessed at 3 day intervals (3,6,9,12 days) by measuring quantities of sGAG released into culture media and remaining on culture substrates. Media from culture samples (3 wells per substrate condition for each of n = 3 experiments) was collected following 3 day culture intervals and stored at −20°C until the assay. Cells and substrate proteins (including BME gel) remaining in sample wells after removal of media were digested in papain solution (300 µg/mL in PBS with 5 mM EDTA and 5 mM cysteine, 65°C for 3 hours), vortexed, and stored at −20°C. Samples from control wells which contained no cells (but included substrates) were collected and processed similarly. sGAG content was measured by mixing samples with DMMB dye and measuring absorbance (535 nm) on a plate reader (Tecan Genios, Mannendorf, Switzerland), with sGAG concentrations calculated from a standard curve prepared from commercial chondroitin-4-sulfate (Sigma). Sample concentrations (in media plus cell digest, corrected using readings from appropriate control samples) were multiplied by the sample volume to yield total sGAG, which was then normalized to DNA content (Quant-iT PicoGreen dsDNA Kit, Invitrogen) for each sample. Differences in sGAG production (sGAG/DNA) amongst culture substrates over time in culture were detected via two-factor ANOVA (substrate, time) and Tukey's HSD post hoc analysis.

## Results

### Mechanical characterization of BME and polyacrylamide gel substrates

**Figure 1:**
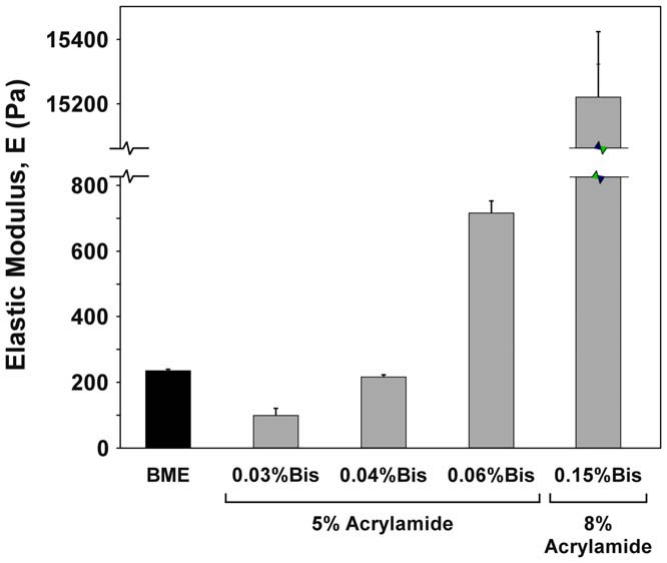
Mechanical properties of basement membrane extract (BME) and polyacrylamide gel substrates. Elastic moduli (mean ± SD) of polymerized BME and acrylamide gel substrates (5% or 8% acrylamide with varying bis-acrylamide crosslinker concentrations) measured via atomic force microscopy (AFM) indentation (5 µm spherical-tipped indenter, n = 75 indentation tests/gel formulation), p<0.0001 for all comparisons except BME vs. 0.04% Bis (p = 0.635).

The elastic moduli of self-polymerizing BME and various formulations of polyacrylamide gel substrates were measured via AFM indentation. The BME gel modulus was measured to be E_BME_ = 235±5 Pa, as shown in Figure 1. Polyacrylamide gel formulations (5% or 8% acrylamide, 0.03%–0.15% bis-acrylamide) yielded gels with elastic moduli ranging from 100±18 Pa to 15200±197 Pa (Figure 1), providing substrates with elastic moduli that were similar to, as well as stiffer than, that measured for self-polymerizing BME. Statistically significant differences existed for all comparisons (p<0.0001), except between BME and 0.04% bis substrates (p = 0.635). Polyacrylamide gel formulations were found to produce uniform surfaces (mean thickness = 122±9 µm, n = 8 gels). AFM indentation curves were found to correlate closely with the Hertz model for a spherical indenter (minimum R^2^ value of 0.98 for all gels), and variation was small, with standard deviations typically less than 5%. No difference in stiffness was detected between blank polyacrylmide gels and those functionalized with BME ligand (2104±57 Pa and 2114±192 Pa, respectively; p = 0.67).

### NP cell-cell interactions on BME gel substrates

**Figure 2:**
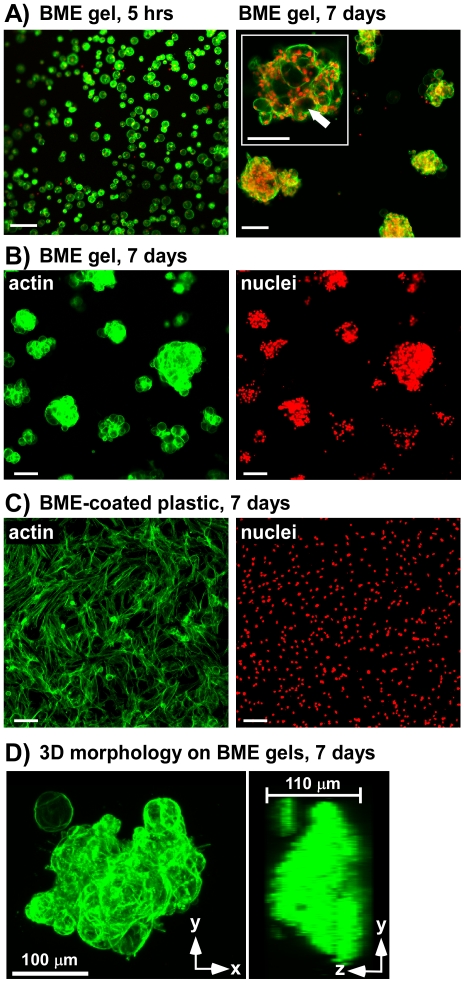
NP cell organization on laminin-rich basement membrane extract (BME) substrates. (A) NP cells seeded on soft BME gel substrates attach as individual cells (left) and self-assemble into cell clusters (right) following 7 days culture (cells stained with Alexa 488 phalloidin to label actin (green) and propidium iodide to label cell nuclei (red)). A confocal section (7 µm thick image slice) of an NP cell cluster (right, inset) shows cell voids (white arrow) consistent with intracellular vacuoles. (B) NP cell clusters (7 days culture) showing actin cytoskeleton (left) and cell nuclei (right). (C) NP cells (7 days culture) on unpolymerized BME-coated rigid tissue culture plastic (left: actin, right: nuclei). (D) Confocal image stack projections (left: in plane with gel surface; right: perpendicular to gel surface) of actin cytoskeleton illustrating 3D nature of cell clusters. Scale bars = 100 µm.

NP cells seeded onto self-polymerizing 2D BME gel substrates were found to attach as individual cells (Figure 2A, left), with 95% of attached cells present as single cells after 5 hours of culture. Cells remained spherical following attachment (Figure 2A), with no indication of cell spreading on gel surfaces. Over 7 days of culture, NP cells were found to reorganize to form large multi-cell clusters (Figure 2A right, 2B), with almost all cells (98%) present in clusters. Clusters were large in size (115±100 cells/cluster, see Table S1 for cluster dimensions) and 3-dimensional (often greater than 5 cell layers high, Figure 2D). Confocal image sections of clusters stained for actin and cell nuclei revealed an interconnected network of cells, with large void areas consistent with intracellular vacuoles (Figure 2A, inset). This morphology and cellular organization is reminiscent of *in situ* NP cell morphology observed in immature NP tissues [12], [46]. In contrast to BME gel substrates, NP cells seeded on tissue culture plastic coated with unpolymerized BME formed a uniform monolayer (Figure 2C), with cells exhibiting an elongated, flattened morphology with dense stress fibers. No cell clustering was observed, with cell nuclei uniformly distributed (Figure 2C, right).

### ECM stiffness and ligand modulate NP cell-cell interactions

**Figure 3:**
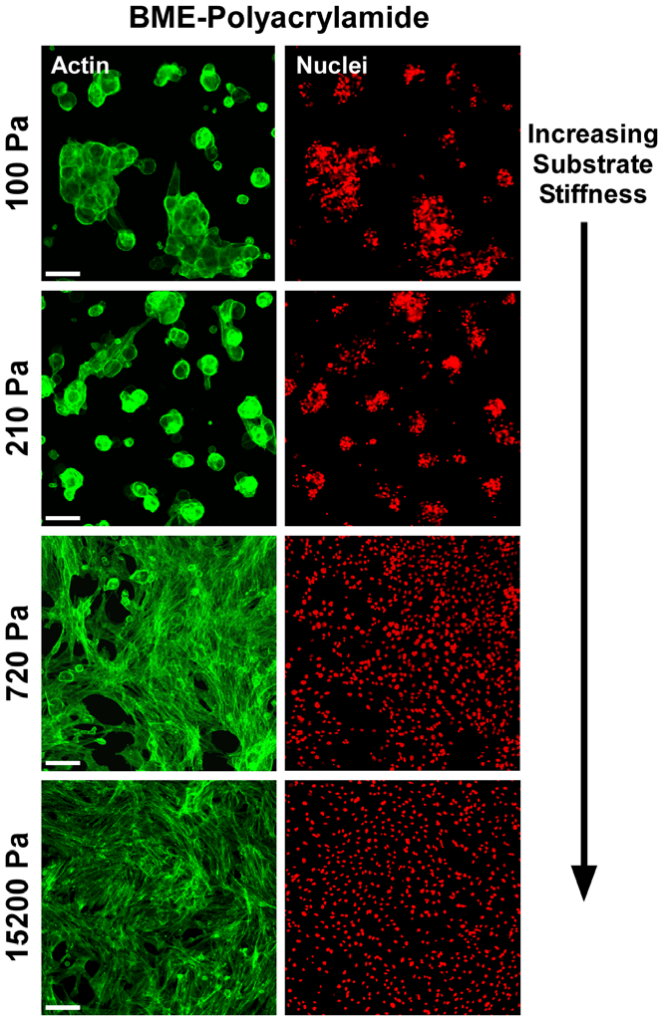
Effect of substrate stiffness for NP cells cultured on BME-functionalized gel substrates. NP cells assemble into multi-cell clusters on soft (100 Pa, 210 Pa), but not stiff (720 Pa, 15,200 Pa), polyacrylamide gel substrates functionalized with BME ligand. Representative images of NP cell organization and morphology (green: actin; red: cell nuclei) following 7 days of culture are shown. Scale bars = 100 µm.

**Figure 4:**
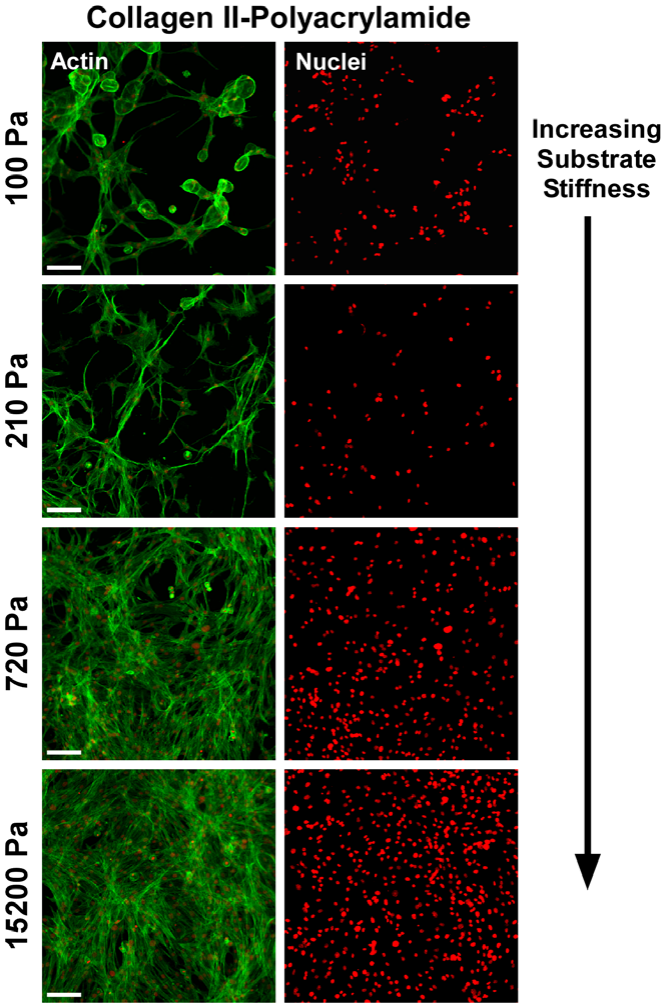
Effect of substrate stiffness for NP cells cultured on collagen-functionalized gel substrates. NP cells do not form multi-cell clusters on polyacrylamide gel substrates functionalized with type II collagen, regardless of stiffness. Representative images of NP cell organization and morphology (green: actin; red: cell nuclei) following 7 days of culture are shown. Scale bars = 100 µm.

**Figure 5:**
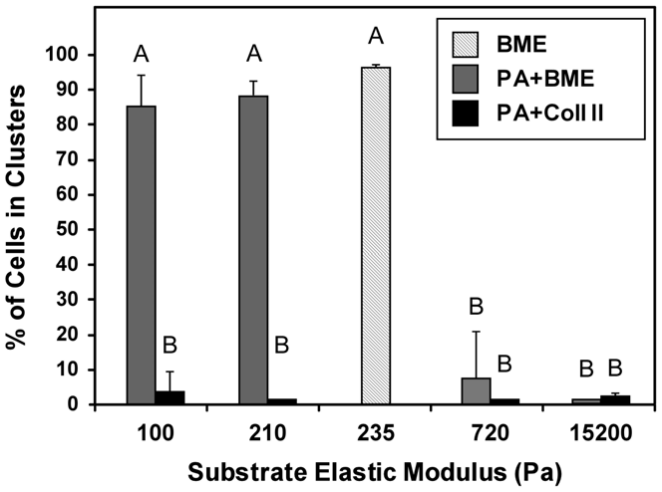
Percentage of NP cells present in multi-cell clusters. Cells were cultured 7 days on basement membrane extract (BME) gels and polyacrylamide (PA) gel substrates functionalized with either BME (PA+BME) or type II collagen (PA+Coll II). Data are shown as mean ±SD. A significant difference was detected for conditions labeled with different letters (2-factor ANOVA, Tukey's HSD, p<0.0001).

To evaluate the specific roles of ECM ligand and substrate stiffness in the observed NP cell-cell interactions, we examined cell clustering behavior on mechanically-tunable polyacrylamide gels functionalized with either unpolymerized BME (Figure 3) or type II collagen (Figure 4) ligands. On BME-functionalized gels with stiffnesses similar to or less than E_BME_ (i.e. 100 Pa, 220 Pa), distinct clustering behaviors were observed following 7 days culture (Figure 3, top 2 panels), with >90% of all cells present in clusters on 100 Pa and 210 Pa gels (Figure 5). These clusters were smaller in size (cell number, maximum dimension, and height) than clusters on BME gels (Table S1), but were still 3-dimensional (typically 2–3 cell layers high). Actin staining revealed a cortical arrangement with very few stress fibers or elongated cells. In contrast, NP cells on somewhat stiffer BME-functionalized gels (720 Pa, Figure 3) exhibited dramatically less clustering, with just 10% of cells present in clusters (Figure 5). Cells on this gel stiffness primarily formed a monolayer sheet (<25 µm height), with elongated cell morphologies and numerous actin stress fibers (Figure 3). Similar behavior was observed on the stiffest gel substrates (Figure 3), with no cell clusters observed (0%, Figure 5).

Clustering behaviors of NP cells on collagen-functionalized acrylamide gels (Figure 4) were notably different than on BME, with little cell clustering behavior observed at even the lowest substrate stiffnesses. Just 5% of NP cells were present in clusters on the softest collagen substrates (100 Pa, Figure 4), and less than 1% of cells on stiffer collagen-functionalized substrates were found in clusters (220–15,200 Pa). On the softest substrates (100 and 220 Pa), cells assumed a spindle-shaped morphology, with long, thin projections extending from cell bodies (Figure 4). As substrate stiffness increased (>100 Pa), cells were found to form a dense monolayer and contained numerous actin stress fibers. Together, these studies utilizing mechanically-tunable polyacrylamide gels indicate that the NP cell-cell organization is a function of both ECM ligand and stiffness, requiring soft (<720 Pa), BME-functionalized substrates to promote NP cell-cell interactions.

### Mechanical characterization of IVD cells

**Figure 6:**
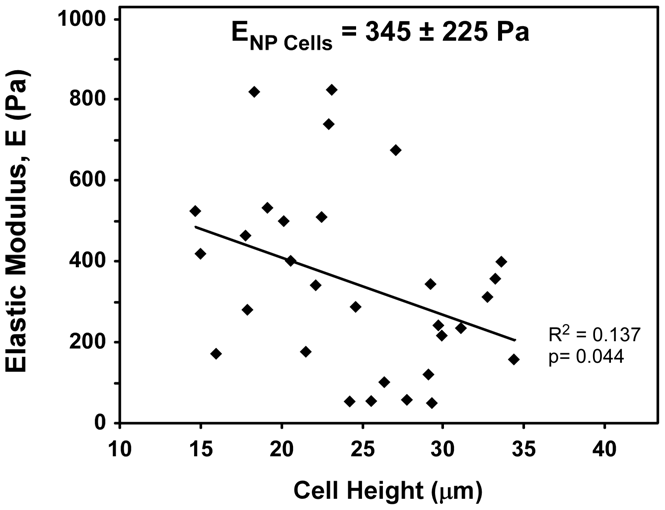
NP cell elastic modulus and cell height measured via atomic force microscopy (AFM) indentation. The mean elastic modulus of freshly isolated NP cells (n = 30) was found to be E_NP Cells_ = 345±245 Pa. NP cell height (mean height = 24.7±5.8 µm) was found have a slight negative correlation with elastic modulus (slope of a linear fit significantly different from zero, p = 0.044).

To understand how the mechanical properties of individual NP cells compared to those of culture substrates, the elastic moduli of freshly isolated NP cells were measured via AFM indentation. NP cells (n = 30) were found to have an average elastic modulus of E_NP_Cells_ = 345±225 Pa (Figure 6); within this NP cell population, there was a fairly large range of moduli, varying from 49 Pa to 825 Pa. NP cells were found to have a mean cell height of 24.7±5.8 µm, and there was a slight but statistically significant negative correlation between NP cell height and stiffness (Figure 6, linear fit, p = 0.043, R^2^ = 0.137), with taller (i.e. larger) NP cells having lower elastic moduli.

### Roles of ECM stiffness and ligand on NP cell sGAG production

**Figure 7:**
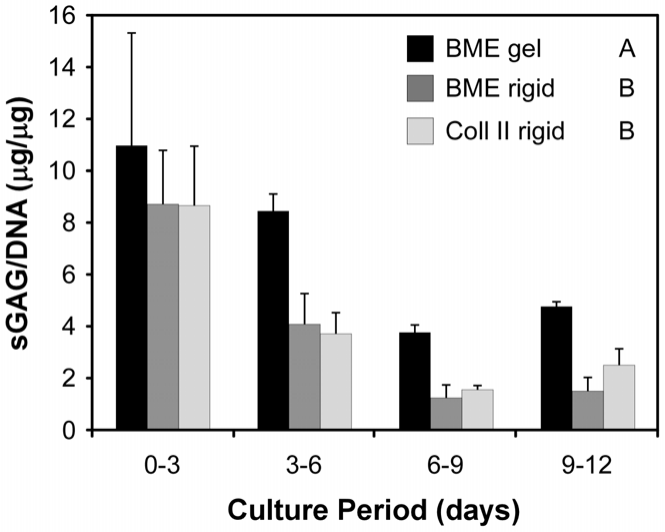
NP cell sulfated glycosaminoglycan (sGAG) production (normalized to DNA content) over time in culture. NP cells were cultured on soft basement membrane extract (BME) gels or ligand-coated tissue culture plastic (BME or type II collagen). NP cells cultured on BME gels produced significantly higher levels of total sGAG (in media plus cell digest) as compared to rigid substrates with either ligand (mean ± SD, n = 3, 2-factor ANOVA with Tukey's HSD; a significant difference was detected between substrates labeled with different letters, p<0.0001). A significant effect of culture period was also detected, with Days 0–3>Days 3–6>Days 6–9 and 9–12 (p<0.0001).

NP cells cultured on soft BME gel substrates were found to produce proteoglycan (sGAG/DNA) at significantly higher levels than NP cells cultured on rigid substrates (p<0.0001), as shown in Figure 7. This difference was apparent for each 3-day culture period, and over 12 days of culture, cells on BME gels produced sGAG at levels approximately 1.7 times greater than cells cultured on rigid BME- or collagen-coated plastic. No difference was detected between rigid substrates coated with different ECM ligands (unpolymerized BME, type II collagen), with cumulative sGAG production on these two substrates almost identical. Analysis of variance also detected a significant effect of culture period (p<0.0001), with the highest quantities of sGAG produced in days 0–3, then progressively decreasing and leveling off (Figure 7; days 0–3>days 3–6>days 6–9, days 9–12). Cell growth patterns (DNA content) over the 12-day culture period were similar for all three substrate conditions (data not shown). Overall, culture on soft BME substrates promoted significantly higher levels of NP cell proteoglycan production as compared to rigid culture substrates.

## Discussion

This study provides new information on the role of the ECM environment on immature NP cell behaviors, including cell organization and phenotype. Upon attachment to soft, laminin-rich BME gel surfaces, NP cells showed minimal spreading and assembled into multi-cell aggregates over 7 days, with practically all cells (98%) found in large clusters. It was notable that even on 2-dimensional gel substrates, 3-dimensional clustering and morphologies were typically observed, with cells forming aggregates which were often greater than 5 cell layers high. These findings suggest that NP cells favor cell-cell interactions over cell-substrate interactions under these specific ECM conditions. This corresponds with the results of several studies examining the balance between cell-cell and cell-substrate adhesions for other cell types, where cell-cell interactions were promoted by reducing substrate stiffness [29], [32], [49]. In contrast to BME substrates, NP cell behaviors on collagen substrates were markedly different, with cells having spindle-like morphologies with long, thin processes extending away from or between cells. On the softest collagen substrates (100 Pa), NP cells remained spindle-like and did not form discrete clusters, although end-to-end cell networks were observed which appear similar to those reported for endothelial cells on soft, collagen-functionalized substrates [49].

The findings for increased cell-cell interactions and tissue-like cellular organization on (or within) soft BME substrates have been well-studied for several epithelial cell types, including mammary epithelial cells (MECs) [50], [51], [52]. These epithelial cells form polarized, functional cell clusters in response to a 3-dimensional BME culture environment [53] and share several phenotypic and *in situ* organizational characteristics with immature NP cells, including strong cell-cell adhesions [10], [11] and laminin cell-ECM adhesions [13], [36], [37], [42]. In that culture system, two signals provided by the culture environment are critical for promoting organization and phenotypic maintenance: (1) the presence of the laminin isoform LM-111 [54], [55] and (2) soft (<400 Pa) substrate elasticity [31]. These findings correspond closely with the results of the present study, as immature NP cells required both BME ligand and soft (100–220 Pa) substrate stiffnesses for clustering behaviors. The embryonic notochord has been described as a “primitive epithelia” [10], and the similarities in organizational behaviors observed in this study may be further evidence that characteristics of this epithelial-like phenotype are preserved in notochordally-derived immature NP cells.

In this study, a polyacrylamide gel system was utilized to investigate the effects of matrix mechanical properties on NP cell behaviors, with substrate stiffness altered by changing the crosslinker concentration of the gel. Altering crosslinker concentration may alter other gel properties in addition to mechanical stiffness, including gel porosity and ligand density, and these factors could also contribute to the differences in cell behaviors observed. Previous studies using the same polyacrylamide system have reported that the ligand density presented to the cell is similar across gels of varying stiffness and porosity [44], [56], however, suggesting that variable ligand density may not have been a large influencing factor in results presented here. Furthermore, we used ligand functionalizing concentrations in the current study that were far above the minimum required for cell adhesion (2 to 4-fold,data not shown), in an effort to avoid low densities where cells may be particularly sensitive to small alterations in ligand concentration. It is also noted that some differences in cluster size and cell morphology (Figures 2 and 3, Table S1) were observed between BME gels and BME-functionalized acrylamide gels with comparable stiffnesses (E = 100, 220 Pa), with polyacrylamide gels exhibiting clusters of smaller size. These observations suggest that soft, BME-functionalized polyacrylamide gels did not exactly mimic the ECM environment of self-polymerizing BME gels. These differences could be due to differences in ligand density mentioned above, or could also reflect differences in time-dependent mechanical behavior of the two different types of gels, as polyacrylamide gels have been reported to be primarily elastic in nature [44], [57], whereas BME gels may exhibit significant viscoelastic behaviors [58].

On BME gel substrates, NP cells were found to produce high levels of sGAG relative to other 2D rigid substrates. The specific mechanism which modulates this NP cell phenotypic response is not known, but could potentially involve a role for direct ECM ligand binding which is translated directly into sGAG-producing intracellular cell signaling events via ECM ligand-receptor interactions (e.g. integrins, focal adhesions). An alternate explanation for the elevated sGAG on the BME gel substrate could be that both ECM ligand and substrate stiffness may (individually or in combination) modulate cellular behaviors such as cell shape and cell-cell interactions, which in turn may affect cell phenotype. A more definitive interpretation would require additional experiments of ligand immobilized on polyacrylamide gels of varying stiffnesses, an experiment that was not pursued here. For chondrocytes, it is well known that maintenance of a rounded shape by 3D culture (pellet culture, encapsulation in hydrogels [59], [60]), or by culturing on 2D substrates with low adhesivity or at high cell densities [61], [62], will promote production of cartilage matrix, including increased proteoglycan expression . Similarly, homotypic cell-cell interactions which occur during mesenchymal condensation and chondrogenesis lead to chondrocyte differentiation and upregulate matrix production [63], [64]. In the present study of immature NP cells, it is noted that the largest differences in sGAG production between gel and rigid substrates occurred after Day 3 (production on BME gel was 25% greater than rigid substrates for Days 0–3, but 100–200% greater for Days 3–12, as shown in Figure 7). Morphologically, this corresponded with the time period when NP cells on rigid substrates had begun to spread and deviate from a rounded cell shape and also when NP cells on soft BME substrates had begun to form multi-cell clusters. Thus, peak fold-differences in sGAG production between BME gel and rigid substrates appear to coincide with time periods where cell shape differences and cell-cell interactions had been maximized.

Our investigation of the mechanical properties of immature NP cells revealed their stiffness (E_NP_Cells_ = 345 Pa) to be similar to, or greater than, the magnitude of (BME-functionalized) substrate stiffnesses where NP cells preferred an aggregating morphology (100 Pa, 220 Pa). This finding may corroborate previous work by Guo and colleagues [32], who observed that cells from rat cardiac tissue would migrate out of soft tissue onto substrates which were stiffer than, but not softer than the native tissue. They (and others [29]) have proposed that cells may seek to maximize mechanical input (i.e. generate maximum traction) from their environment, via either cell-substrate or cell-cell interactions. However, the findings that NP cell clustering was much more prevalent on BME-functionalized surfaces than on collagen-functionalized substrates of the same stiffness suggests that chemical cues (i.e. ligand signaling or adhesivity) are also critical in determining whether a clustering morphology occurs. The magnitude of E_NP_Cells_ differs somewhat from the findings by Guilak and co-workers [65], wherein micropipette aspiration experiments porcine NP cells were found have an instantaneous modulus of approximately 800 Pa. Additionally, Guilak and co-workers found a positive correlation between NP cell size and equilibrium elastic modulus; in the present study, a (weak) negative correlation was detected. These discrepancies may be a result of the different testing methods employed, with micropipette aspiration and cell indentation applying different types of loading (tensile and compressive, respectively) to the cell. In addition to testing method, differences in culture conditions prior to testing may also have been critical: for micropipette studies, cells were dissociated, suspended in alginate beads and cultured for 1–3 days prior to testing; in the present study, cells were tested within 3 hours of dissociation. Furthermore, micropipette aspiration tests cells in suspension, whereas AFM tests cells adhered to a substrate. Despite these differences, the average NP cell modulus in both studies fell within a fairly narrow range (350–800 Pa).

There are several possible implications for the findings of NP cell-cell interactions documented here. NP cell organization *in vitro* was shown to be highly sensitive to substrate stiffness, where an increase in substrate elastic modulus of ∼500 Pa resulted in an almost complete loss of cell clustering behavior. As the stiffness of NP tissue has been documented to increase with increasing age and degeneration [66], it is possible that alterations in tissue stiffness could be a contributing factor in dissociation, differentiation, or cell death of clustered immature NP cells. Likewise, clustering behaviors were only observed on BME-functionalized substrates, suggesting that changes in ECM ligand environment (e.g. decreased presence of laminin ligands) could also play a role in aging-related changes in NP cell organization or phenotype. Finally, the ECM ligand and substrate stiffness findings presented here may be useful for developing tissue engineering strategies for the NP, as BME substrates of an appropriate stiffness were found to promote immature NP cell proteoglycan synthesis. Future work focused on NP cell biological responses to soft laminin biomaterials is necessary to further elucidate the role of the ECM environment in regulating NP cell survival, metabolic activity, and phenotype.

In this study, soft, laminin-rich BME culture substrates were found to promote immature NP cell-cell interactions *in vitro*, with NP cells exhibiting clustered cell organization and morphologies similar to those observed *in situ*. These clustering behaviors were identified to be a function of both ECM ligand and substrate stiffness, with NP cells shifting from highly clustered (>90% clustered) to monolayer (<10% clustered) with increasing substrate stiffness; in contrast, little or no clustering behavior was observed on collagen substrates of any stiffness. ECM culture environment was also found to affect a key measure of NP cell function, proteoglycan production, with NP cells cultured on soft BME substrates producing 70% more sGAG over a 12 day culture period as compared to culture on other 2-dimensional substrates. Together, these findings suggest that immature NP cell organization and phenotype are highly sensitive to their ECM environment, and that alterations in either of these ECM parameters (stiffness, ligand) during maturation and aging could affect NP cell organization, function, or fate.

## Supporting Information

Table S1 **NP cell cluster sizes on BME and BME-functionalized acrylamide gel substrates.** Mean ± SD shown for each measurement, ≥25 clusters per condition. For each measurement (columns), substrates not labeled with the same letter were statistically different (1-factor ANOVA (substrate) with Tukey's HSD, p<0.005).
(DOCX)
