# Supplementary material for: Extracellular Matrix Ligand and Stiffness Modulate Immature Nucleus Pulposus Cell-Cell Interactions
Source: PLoS One. 2011 Nov 7;6(11):e27170. doi: 10.1371/journal.pone.0027170 (PMC3210142; doi:10.1371/journal.pone.0027170)
Supplement: Table S1 — NP cell cluster sizes on BME and BME-functionalized acrylamide gel substrates. Mean ± SD shown for each measurement, ≥25 clusters per condition. For each measurement (columns), substrates not labeled with the same letter were statistically different (1-factor ANOVA (substrate) with Tukey's HSD, p<0.005). (DOCX) [file pone.0027170.s001.docx]

**Supporting Information**

Table S1: NP cell cluster sizes on BME and BME-functionalized acrylamide gel substrates. Mean ± SD shown for each measurement, ≥25 clusters per condition. For each measurement (columns), substrates not labeled with the same letter were statistically different (1-factor ANOVA (substrate) with Tukey’s HSD, p<0.005).

| **Substrate** | **Cell Number** | **Height (µm)** | **Max. Dimension (µm)** |
| --- | --- | --- | --- |
| BME gel | 115 ± 100 ^A^ | 99 ± 27 ^A^ | 185 ± 81 ^A^ |
| PA-BME (100 Pa) | 41 ± 80 ^B^ | 53± 16 ^B^ | 143 ± 106 ^A,B^ |
| PA-BME (230 Pa) | 32 ± 40 ^B^ | 45 ± 22 ^B^ | 108 ± 43 ^B^ |
